# Effects of Traditional Chinese Fitness Exercises on Negative Emotions and Sleep Disorders in College Students: A Systematic Review and Meta-Analysis

**DOI:** 10.3389/fpsyg.2022.908041

**Published:** 2022-07-04

**Authors:** Tingting Yang, Yingjie Guo, Yang Cheng, Yue Zhang

**Affiliations:** School of Sports and Human Body Science, Shenyang Sport University, Shenyang, China

**Keywords:** traditional Chinese fitness exercises, negative emotions, sleep disorders, college students, meta-analysis

## Abstract

**Background:**

The purpose of this study was to systematically review the effectiveness of regular traditional Chinese fitness exercises on negative emotions and sleep disorders in college students, and to provide evidence-based evidence and new ideas for the negative emotions and sleep disorders among the college students.

**Methods:**

A systematic search using 5 English (PubMed, Embase, Scopus, EBSCO, and the Cochrane Library) and 4 Chinese (CNKI, WanFang, VIP, and CBM) databases were initiated to identify randomized controlled trials (RCT) assessing the effect of traditional Chinese fitness exercises on negative emotions and sleep disorders among college students. Standardized mean differences (SMD) and their 95% confidence intervals (*CI*) were used to determine the pooled effect of the intervention. The Cochrane bias risk assessment tool was used to evaluate the methodological quality and the data were analyzed with Review Manager 5.4.

**Results:**

A total of 12 RCTs were included, including 1,052 subjects. The results showed a potential beneficial effect of traditional Chinese fitness exercises on reducing depression [SMD = −0.93, 95 %*CI* (−1.76, −0. 10)], anxiety [SMD = −0.74, 95%*CI* (−0.93, −0.54)], and the sleep disorders [SMD = −2.77, 95%*CI* (−4.57, −0.97)] symptoms, and these effects were better than in the control group.

**Conclusion:**

The findings of this review suggested the traditional Chinese fitness exercises could improve both the negative moods and sleep disorders compared with that of healthy students, the effect on college students with mild to moderate psychological symptoms was obviously improved. The SCL-90 scale is better than the SDS scale in evaluating the improvement effect of traditional Chinese fitness exercises on depression. It was the best intervention program on negative emotions and sleep disorders among college students, with the intervention lasting 5 sessions per week for 30–60 min/session for over 12 weeks.

## Background

Stress-associated negative emotions and sleep disorders in college students are serious problems that can affect academic outcomes as well as the overall well-being of the student (So et al., 2019). In a DATE mental health survey among 126,000 Chinese college students, approximately 20.23% of the participants previously experienced, or were currently experiencing, different degrees of stress-induced psychological disorders. Of these, anxiety, depression, and sleep disorders accounted for the largest proportion (Jiao et al., 2021). Currently, legally prescribed medication and psychological counseling are the two best treatment methods for college students' psychological illnesses; however, the negative side effects associated with these treatments such as high dependence (Haack et al., 2013) as well as high costs have prevented students from seeking help or continuing treatment. Therefore, many students give up treatment, leading to an increased prevalence of psychological illness year by year.

China's national traditional fitness exercises are a set of fitness systems with oriental characteristics, such as qigong, healthy preservation, martial arts, and swordsmanship, etc. (Yeung et al., 2018). These exercises have been practiced for over 4,000 years. The goal of these exercises was, and is, to promote longevity and health (Jahnke et al., 2010). The main traditional Chinese fitness exercises currently practiced includeTai Chi(太极), Baduanjin(八段锦), Yijinjing(易筋经), Wuqinxi(五禽戏), Liuzijue(六字诀), Dunqianggong(蹲墙功), Zhanzhuanggong(站桩功), etc. These exercises are vigorously promoted by the Qigong Center of the General Administration of Sports of the People's Republic of China and are safe, effective, economical, and environmentally-friendly (Sun et al., 2022; Tong, 2022). Because of this, these traditional exercises are very popular among people of all ages in China. In recent years, the quantitative effect of these exercises on health has been gaining more focus, and thus, more active practitioners. For example, individuals who practice Qigong or Tai-Chi do so primarily to promote physical and psychological wellbeing and to treat various health conditions (Toneti et al., 2020). Thus, traditional Chinese fitness exercises have been increasingly used as complementary and alternative therapies to manage psychological stress and mood disorders (Trkulja and Barić, 2020). In China, traditional Chinese fitness exercises have been incorporated into college physical education courses, promoting the physical and mental health development of Chinese college students. The inclusion of such exercises has been welcomed and enjoyed by thousands of college students (Zou et al., 2017; Guo et al., 2018).

The limbic system of the human brain is responsible for emotion regulation. Specifically, it is responsible for controlling the function of the hypothalamic-pituitary-adrenal (HPA) axis. Preliminary studies have shown that traditional Chinese fitness exercises that focus on a slow breathing rate can reduce the HPA axis activities that are related to stress, adjust the balance of the autonomic nervous system, and enhance parasympathetic functions to improve insomnia, anxiety, depression, and other negative moods (Yeung et al., 2018). As traditional Chinese fitness exercises can reduce the degree of muscle tension, skin electricity, heart rate, and breathing rate, and improve the brain wave α level and the physical and mental problems of anxiety, compulsion, pain, and mood improvement, many studies have suggested that they should be used as an alternative and complementary therapy (Jahnke et al., 2010). Together these data imply that traditional Chinese fitness can alleviate the symptoms of depression, anxiety, and insomnia in college students. Additionally, previous studies have shown that traditional Chinese fitness exercises can increase human melatonin levels and enhance the cell function of granulocytic neutrophils and natural killer cells in the blood, thus improving the human sleep quality as well (Cui and Bai, 2014).

Based on the positive effects of traditional Chinese fitness exercises such as Tai Chi(太极), Baduanjin(八段锦), Yijinjing(易筋经), Wuqinxi(五禽戏), Liuzijue(六字诀), Dunqianggong(蹲墙功), Zhanzhuanggong(站桩功), on the mental health of college students (Lu, 2017), many Chinese universities have begun offering an elective courses in Chinese traditional fitness (Ma et al., 2021). These courses were especially popular during the online learning period of the COVID-19 epidemic where college students used these exercises as an effective means to improve their physical and mental health (Zhang, 2020). Research on the use of traditional Chinese fitness exercises to improve the psychological state of college students has achieved certain results (Shuai et al., 2018; Lu and Wang, 2020; Tan and Tan, 2020; Wang et al., 2020), however, many of these studies are confounded by small sample sizes, and low methodological quality as well as different outcome indicators. In this study, a meta-analysis of the current and previously published traditional Chinese fitness literature was used to conduct a comprehensive and quantitative analysis to determine the ability of traditional Chinese fitness exercises to improve negative emotions and sleep disorders in college students. This work further aims to provide scientific guidance for college students' practice behaviors using the traditional Chinese fitness exercises.

## Methods

This meta-analysis was conducted in accordance with the Preferred Reporting Items for Systematic Reviews and Meta- Analyses (PRISMA) guideline (Moher et al., 2009).

### Search Strategy

Total 2 reviewers independently searched the literature using the following English and Chinese databases: PubMed (all years), Embase (all years), The Cochrane Library (all years), Scopus (all years), EBSCO (all years), the Chinese National Knowledge Infrastructure (CNKI, all years), Wanfang (all years), the Chinese BioMedical Literature on disc (CBMdisc, all years), and the Chinese Scientific Journal (VIP, all years). The searches were conducted from inception through October 2021. Discrepancies between the 2 reviewers (ZY, CY) were discussed until consensus was reached, any disagreements regarding the inclusion would be discussed and resolved with the third reviewer (GYJ). The search terms used in this study was based a previous related meta-analysis: Chinese traditional exercise, Qigong(气功), Tai Chi(太极), Baduanjin(八段锦), Yijinjing(易筋经), Wuqinxi(五禽戏), Liuzijue(六字诀), Dunqianggong(蹲墙功), Zhanzhuanggong(站桩功) anxiety, depression, emotion regulation, sleep disorders, college student, randomized controlled trial, clinical trial, RCT. Chinese translations of these terms were used in Chinese databases. A complete record of search strings is provided in the Supplementary Material. A manual search of reference lists of all included studies and relevant reviews was conducted to further identify relevant studies (Supplementary Table 1).

### Inclusion and Exclusion Criteria

#### Types of Studies

Studies had to be randomized controlled trials (RCTs), and the language of studies is unlimited. A study was defined as RCT if the participants were allocated to experimental and control groups randomly. Repeated publications, review, systematic evaluation, conference summary, Meta-analysis, dissertations, narrative studies, observational or qualitative studies were excluded.

#### Types of Participants

Studies focusing on college students with mean age over 18 years old were included. College students of healthy or mild to moderate psychological symptoms and no motor dysfunction were included.

#### Types of Intervention

Studies had to use any type of traditional Chinese fitness exercises as an intervention with comparison, and the control group had to routine lifestyle. Studies integrating traditional Chinese fitness exercises with other forms of intervention or simply using other forms of intervention were excluded.

#### Types of Outcome Measures

The outcome indicators of studies were related scales that measure the effect of traditional Chinese fitness exercises on negative emotions (e.g., anxiety, depression) and sleep disorders in college students.

### Study Selection and Data Extraction

A total of 2 reviewers independently screened the studies based on the titles, abstracts, and full texts. Discrepancies between the 2 reviewers (ZY, CY) were discussed until consensus was reached, any disagreements regarding the inclusion would be discussed and resolved with the third reviewer (GYJ). 2 reviewers extracted and summarized the following relevant data from all the original articles: (1) the basic characteristics of included studies (i.e., authors, study design, publication date, and country where the trial was performed), (2) the basic characteristics of participants (i.e., mean age, gender, number, sample size, and psychological status), (3) study intervention and comparison group (the types of exercise, single intervention time, frequency, and duration), and (4) outcome parameters.

### Quality Assessment

A total of 2 reviewers independently assessed the risk of bias of all the included studies using the Cochrane Risk of Bias Assessment Tool (Higgins et al., 2011), which includes the following 7 domains: (1) random sequence generation, (2) allocation concealment, (3) blinding of participants and personnel, (4) blinding of outcome assessment; (5) incomplete outcome data, (6) selective reporting, and (7) other biases (Higgins et al., 2011). There are 3 grades for each domain: low risk of bias, unclear risk of bias, and high risk of bias. Basically, the possibility of various bias is small, the methodology quality is A; partially meets the standard, the methodology quality is B; completely does not meet the standard, the possibility of bias is high, the methodology quality is C (Chen et al., 2018). Any disagreements regarding the risk of bias assessment would be discussed and resolved with the third author.

### Data Analysis

The Review Manager 5.4 was applied to perform the meta-analysis. The intervention effect size in each study was presented by the standardized mean differences (SMD) with 95% confidence intervals (CI). Use of SMD allows for the comparisons across included studies where they used different psychometric instruments to measure the same outcome (Deeks et al., 2008; Orwin and Vevea, 2009). The included studies were anticipated to be heterogeneous because of the different characteristics of intervention and control types. To account for the potential heterogeneity, a random-effects model was used throughout data synthesis. I^2^ statistic was used to assess heterogeneity. Studies with an I^2^ statistic of >75% were considered to have a high degree of heterogeneity; studies with an I^2^ statistic of 50–75% were considered to have a moderate degree of heterogeneity; and studies with an I^2^ statistic of <50% were considered to have a low degree of heterogeneity (Higgins et al., 2003). Subgroup analysis on primary outcomes based on intervention plan, psychological healthy level, and scale type was conducted where necessary. In addition, the publication bias was evaluated using the funnel plot asymmetry if 10 trials (at least) were covered in a meta-analysis. Results were considered statistically significant when *p* < 0.05.

### Patient and Public Involvement

No patient involved.

## Results

### Search Results

**Figure 1:**
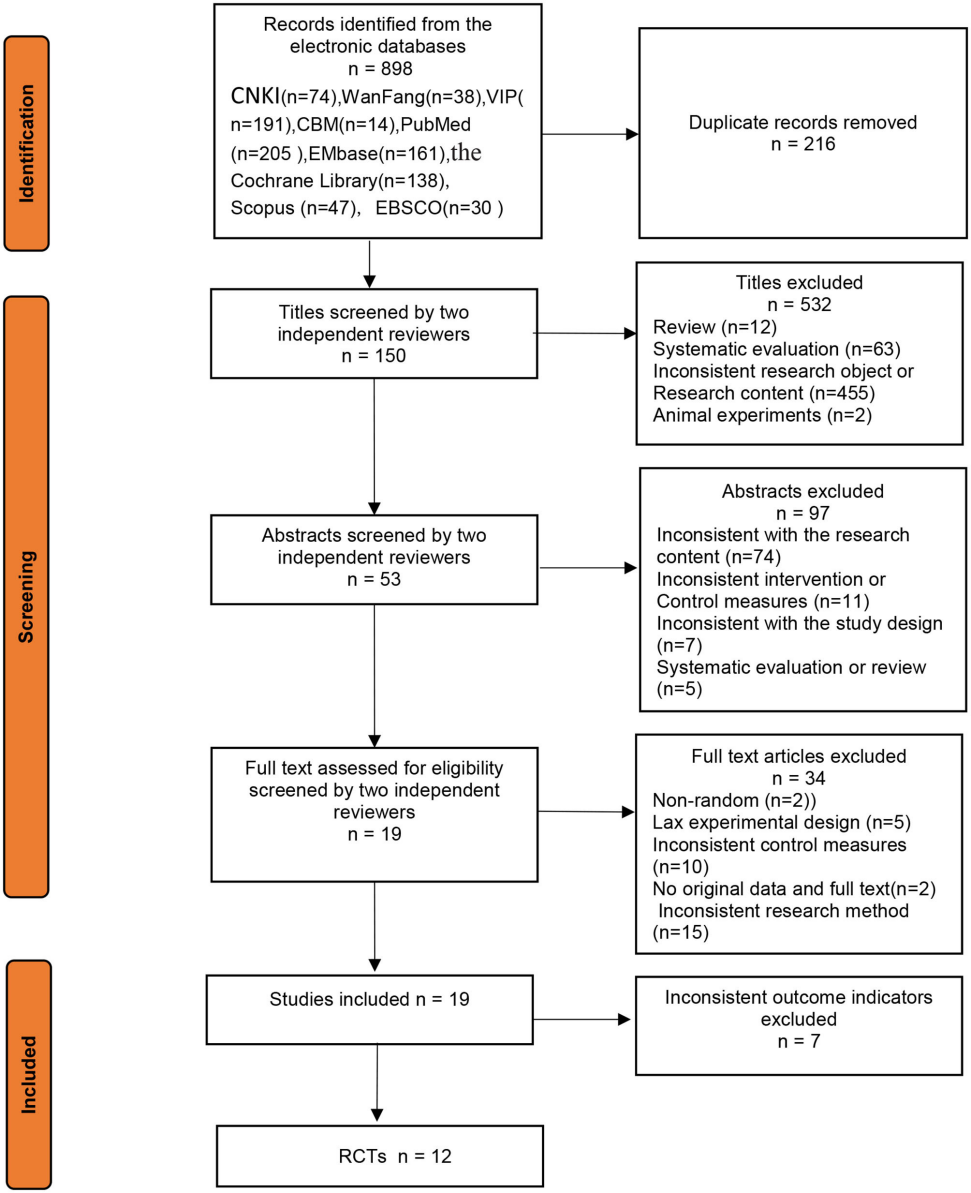
Flow diagram of literature search and selection process.

After searching multiple databases, a total of 821 studies were identified. After removing 671 irrelevant or duplicate records based on titles, the remaining 150 studies were further evaluated according to the following eligibility criteria:(1) Studies with inconsistent intervention or control measures, (2) studies that were inconsistent with the research content, (3) studies that had no original data and full text, and (4) studies that were inconsistent with the study design, reviews, and systematic evaluations, or studies that had an inappropriate sample size were excluded. Using these exclusion criteria, 131 studies were removed prior to analysis. Additionally, 7 studies were excluded due to inconsistent outcome indicators. In total, 12 studies were included for subsequent analyses in this review (Figure 1).

### Description of Included Studies

The basic characteristics of all the included studies are shown in Table 1. In the 12 included RCTs, all of them were conducted in People's Republic of China (*n* = 12, 100%), and 12 studies were published in Chinese. The 12 studies involved a total of 1,052 students aged over 18 years old (516 in the experimental group and 536 in the control group), and all studies (*n* = 12, 100%) included both male and female participants. Among12 studies, Tai Chi (太极) was applied in 3 studies (Rao, 2014; Huang, 2016; Chen et al., 2019), Baduanjin (八段锦) was applied in 2 studies (Su, 2014; Yan and Wei, 2017), Yijinjing (易筋经) was applied in 1 studies (Wei et al., 2017), Wuqinxi (五禽戏) was applied was applied in 2 studies (Cheng et al., 2016; Wang et al., 2020), Liuzijue (六字诀) was applied in 1 studies (Cui and Bai, 2014), Dunqianggong (蹲墙功) was applied in 1 studies (Liu et al., 2017), Zhanzhuanggong (站桩功) was applied in 1 studies (Guo, 2019), more than 2 kinds of traditional Chinese fitness exercises was applied in 1 studies (Zhang, 2021). The psychological status of college students in 5 studies was healthy. In an additional 5 studies, the college students where characterized as having mild to moderate psychological problems. College students in the last two studies were characterized as having sleep disorders. In all of the studies, the practice period ranged from 4 to 24 weeks with single exercise sessions ranging from 30 to 60 minutes. The frequency of practice for all studies ranged from 2 to 7 times per week. 7 scales were used to assess the intervention outcome measures.

**Table 1:** Characteristics of included study (*n* = 12).

**Author (year)**	**Study design**	**Country/ Region**	**Study participants**	**Psychological status**	**Sample size (finished, N) and gender (M/F)**	**Intervention (frequency)**	**Control**	**Relevant outcome (measurements)**
Rao (2014)	RCT	China	College student (mean age TCFE: 20.70 ± 1.10 CON: 20.60 ± 1.20)	Healthy	N: 198 TCFE: 95 33/62 CON: 103 32/71	Tai Chi (12 weeks, 60 min/session, 5 times per week	Wait list	(1)(2)
Huang (2016)	RCT	China	College student (mean age TCFE: 18.68 ± 1.76 CON: 18.67 ± 1.50)	Sleep disorders	N: 50 TCFE: 22 NA CON: 28 NA	Tai Chi (24 weeks, 60 min/session, 5 times per week)	Wait list	(2)(3)
Yan and Wei (2017)	RCT	China	College student (mean age: NA)	Healthy	N: 100 TCFE: 50 NA CON: 50 NA	Baduanjin (12 weeks, 30 min/session, 5 times per week)	Wait list	(1)
Cui and Bai (2014)	RCT	China	College student (mean age: NA)	Sleep disorders	N: 60 TCFE:30 NA CON: 30 NA	Liuzijue (8 weeks, 60 min/session, 5 times per week)	Wait list	(2)
Wei et al. (2017)	RCT	China	College student (mean age: NA)	Healthy	N: 120 TCFE: 60 NA CON: 60 NA	Yijinjing (12 weeks, 30 min/session, 5 times per week)	Wait list	(1)
Su (2014)	RCT	China	College student(mean age:NA)	Healthy	N: 206 TCFE: 101 NA CON: 105 NA	Baduanjin (13 weeks, 60 min/session, 5 times per week)	Wait list	(1)(2)
Liu et al. (2017)	RCT	China	College student (mean age TCFE: 20.27 ± 1.76 CON: 20.84 ± 1.30)	Healthy	N: 62 TCFE: 30 14/16 CON: 32 16/16	Dunqianggong (4 weeks, 30 min/session, 7 times per week)	Wait list	(1)
Wang et al. (2020)	RCT	China	College student (mean age: NA	Mild to moderate psychological symptoms	N: 60 TCFE: 30 NA CON: 30 NA	Wu Qin Xi (24 weeks, 60 min/session, 7 times per week)	Wait list	(4)(2)
Guo (2019)	RCT	China	College student (mean age TCFE: 19.03 ± 0.89 CON: 19.25 ± 0.67)	Mild to moderate psychological symptoms	N: 70 TCFE: 35 NA CON: 35 NA	Zhanzhuanggong (14 weeks, 50 min/session, 5 times per week)	Wait list	(1)
Chen et al. (2019)	RCT	China	College student (mean age: NA)	Mild to moderate psychological symptoms	N: 36 TCFE: 18 NA CON: 18 NA	Tai Chi (16 weeks, 60 min/session, 3 times per week)	Wait list	(5)
Cheng et al. (2016)	RCT	China	College student (mean age TCFE: 21.10 ± 1.40 CON: 21.00 ± 1.60)	Mild to moderate psychological symptoms	N: 30 TCFE: 15 7/8 CON: 15 8/7	Wu Qin Xi (12 weeks, 40–60 min/session, 3 times per week)	Wait list	(6)
Zhang (2021)	RCT	China	College student (mean age: NA)	Mild to moderate psychological symptoms	N: 60 TCFE: 30 NA CON: 30 NA	More than 2 kinds of traditional fitness exercises (12 weeks, 60 min/session, 7 times per week)	Wait list	(4)(7)

*RCT, Randomized Controlled Trial; N, number; M, male; F, female; TCFE, Traditional Chinese Fitness Exercises; CON, Control Group; (1) SCL-90(The Symptom Chcklist-90); (2) PSQI(Pittsburgh Sleep Quality Index); (3) STAI(State Trait Anxiety Inventory); (4) SDS(Self-Rating Depression Scale); (5) CES-D(Center for Epidemiological Studies-Depression);(6) HAMD(Hamilton Depression Scale); (7) SAS(Self-rating Anxietyscale Scale)*.

### Study Quality Assessment

**Figure 2:**
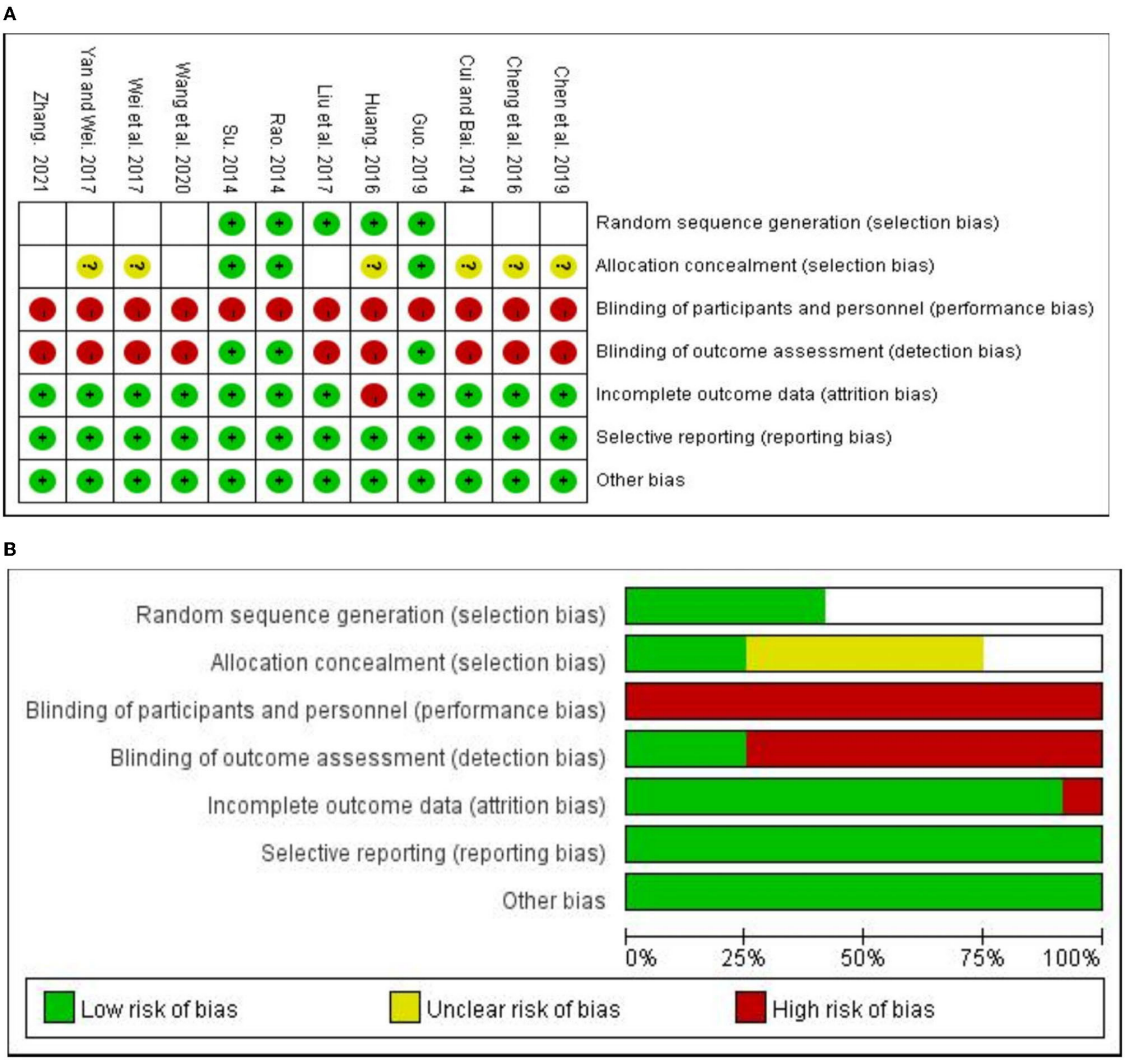
Risk of bias summary **(A)** and graph **(B)**.

Table 2 and Figures 2A,B presents the methodological quality of all included studies. The generation of random allocation was adequately conducted in all study. Among the 12 included RCTs, the studies quality gradeAhad3 studies (Rao, 2014; Su, 2014; Guo, 2019), the studies quality grade B had had 9 studies (Cui and Bai, 2014; Su, 2014; Huang, 2016; Liu et al., 2017; Wei et al., 2017; Yan and Wei, 2017; Chen et al., 2019; Wang et al., 2020; Zhang, 2021), and no grade C. The results showed that the allocation hidden uncertainty of the 12 included studies and the risk bias of the blind setting were relatively high. Regarding the allocation concealment, there were only 3 studies (Rao, 2014; Su, 2014; Guo, 2019) clearly described the allocation concealment. In addition, since there is less trial reported to blind their participants, personnel, and outcome assessors or they did not blind them, these 2 domains were also the major sources that increase the risk of bias. However, a low risk of bias was reported in most studies for the incomplete outcome data (*n* = 11) and selective reporting (*n* = 12), and there were no other biases described in these studies.

**Table 2:** Risk of bias summary.

**Trials**	**Random sequence generation**	**Allocation concealment**	**Blinding of participants and personnel**	**Blinding of outcome assessment**	**Incomplete outcome data**	**Selective reporting**	**Other bias**	**Studies quality grade**
Rao (2014)	Low	Low	High	Low	Low	Low	Low	A
Huang (2016)	Low	Unclear	High	High	High	Low	Low	B
Yan and Wei (2017)	Unclear	Unclear	High	High	Low	Low	Low	B
Cui and Bai (2014)	Unclear	Unclear	High	High	Low	Low	Low	B
Wei et al. (2017)	Unclear	Unclear	High	High	Low	Low	Low	B
Su (2014)	Low	Low	High	Low	Low	Low	Low	A
Liu et al. (2017)	Low	Unclear	High	High	Low	Low	Low	B
Wang et al. (2020)	Unclear	Unclear	High	High	Low	Low	Low	B
Guo (2019)	Low	Low	High	Low	Low	Low	Low	A
Chen et al. (2019)	Unclear	Unclear	High	High	Low	Low	Low	B
Cheng et al. (2016)	Unclear	Unclear	High	High	Low	Low	Low	B
Zhang (2021)	Unclear	Unclear	High	High	Low	Low	Low	B

### Outcomes

#### Effects of Traditional Chinese Fitness Exercises on Depression Among College Students

**Figure 3:**
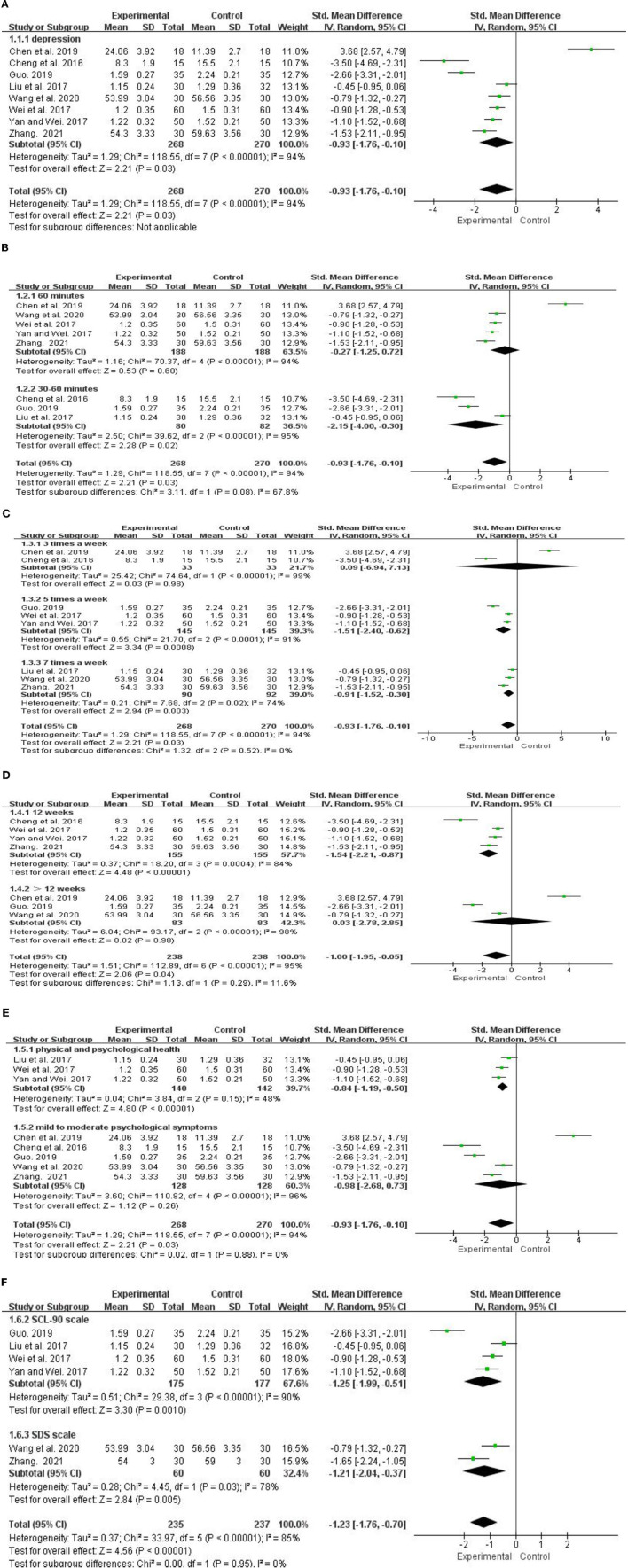
Meta-analysis of traditional fitness exercises on depression **(A)** among college students. CI, condence intervals; IV, inverse variance. Forest plot for subgroup analysis base on single intervention time **(B)**. Forest plot for subgroup analysis base on intervention frequency **(C)**, intervention duration **(D)**, psychological health level **(E)**. Forest plot for subgroup analysis base on scale type **(F)**.

A total of 8 studies (*n* = 538) examined the effect of traditional Chinese fitness exercises on depressive (Cheng et al., 2016; Liu et al., 2017; Wei et al., 2017; Yan and Wei, 2017; Chen et al., 2019; Guo, 2019; Wang et al., 2020; Zhang, 2021). This suggested that the effect of traditional Chinese fitness exercises on reducing depression among college students (SMD =-0.93, 95% *CI* [−1.76, −0.10]), and the depression improvement of experimental group was better than the control group (*p* < 0.05). Heterogeneity among studies was high (I^2^ = 94%). Because of no sources of heterogeneity were found in the sensitivity analysis, so, the subgroup analysis was conducted from 3 aspects of intervention plan (single intervention time, intervention frequency, intervention duration), psychological healthy level, and scale type to further explore the sources of heterogeneity (Figure 3A).

The subgroup analysis based on the intervention plan showed that, the effect size of 30–60 min (SMD = −2. 15, 95% *CI* [−4.00, −0.30]) was higher than the 60 min (SMD = −0.27, 95% *CI* [−1.25, 0.72]) in the single intervention time (Figure 3B). The effect size of 5 times a week (SMD = −1.51, 95% *CI* [−2.40, −0.62]) was the largest in the intervention frequency, effect size of 7 times a week (SMD = −0.91, 95% *CI* [−1.52, −0.30]) followed, and the effect size of 3 times a week (SMD = −0.09, 95% *CI* [−6.94, 7.13]) was the lowest (Figure 3C). The effect size of over12 weeks (SMD = 0.03, 95% CI [−2.78, 2.85]) was higher than 12 weeks (SMD = −1.54, 95% *CI* [−2.21, −0.87]) in the intervention duration. The difference between groups was statistically significant (*p* < 0.05) (Figure 3D). This suggested that the best intervention plan of traditional Chinese fitness exercises to improvement the depressive of college students is practiced over 5 sessions/week for 30–60 min/session for over 12 weeks.

The subgroup analysis based on the psychological healthy level showed that, compared with college students who physical and psychological healthy [SMD = −0.84, 95% *CI*(−1.19, −0.5)], those who mild to moderate psychological symptoms showed higher effect size [SMD = −0.98, 95% *CI* (−2.68, 0.73)] (Figure 3E). The difference between groups was statistically significant (*p* < 0.001). This suggested that the significantly effect of the traditional Chinese fitness exercises on depression in college students with mild to moderate psychological symptoms than in healthy college students.

The depression scales used in this review, included SCL-90 scale (*n* = 4), HAMD scale (*n* = 1), SDS scale (*n* = 2), and CES-D scale (*n* = 1). Because only one study was included in this review using HAMD scale and CES-D scale, the subgroup analysis was not possible, so the scale type was divided into 2 subgroups, namely SCL-90 and SDS. The subgroup analysis based on the scale type showed that, The effect size of the SCL-90 scale [SMD = −1.25, 95% *CI* (−1.99, −0.51)] is higher than the SDS scale [SMD = −1.21, 95% *CI* (−2.04, −0.37)] (Figure 3F). The difference between groups was statistically significant (*p* < 0.001). This suggested that the SCL-90 scale is better than the SDS scale when evaluating the improvement effect of traditional Chinese fitness exercises on depression in college students.

#### Effects of Traditional Chinese Fitness Exercises on Anxiety Among College Students

**Figure 4:**
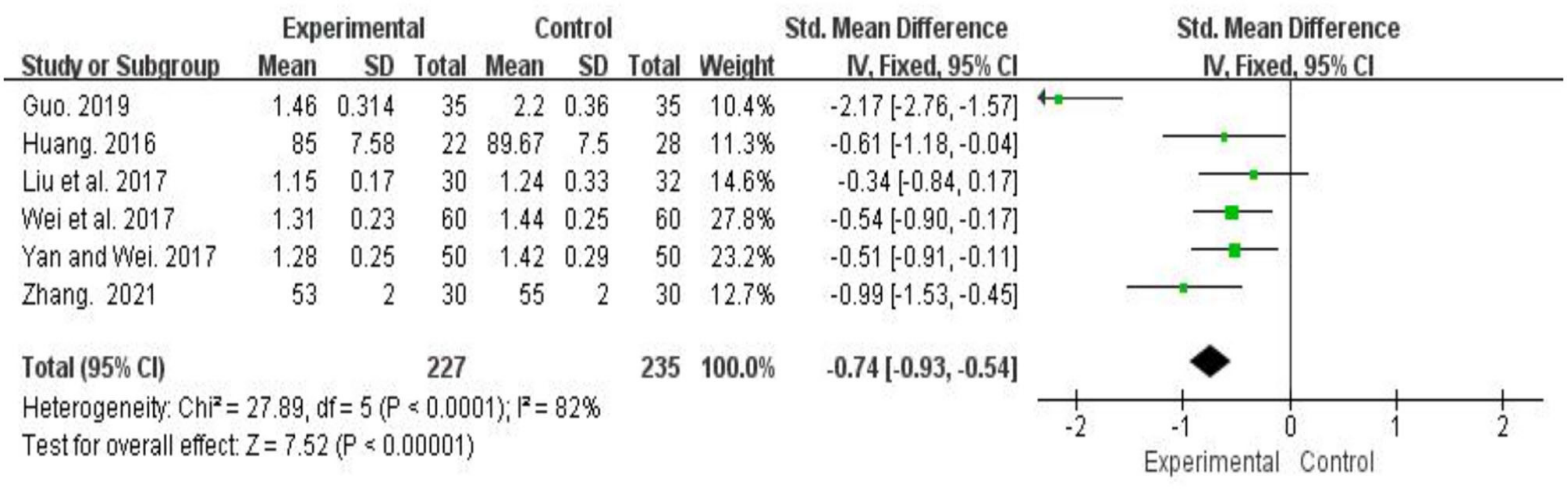
Meta-analysis of traditional fitness exercises on anxiety among college students.

A total 6 studies (*n* = 462) examined the effect of traditional Chinese fitness exercises on anxiety (Huang, 2016; Liu et al., 2017; Wei et al., 2017; Yan and Wei, 2017; Guo, 2019; Zhang, 2021). The pooled results showed that the significantly effect of traditional Chinese fitness exercises on reducing anxiety among college students compared to control group (SMD = −0.74, 95 %*CI* [−0.93, −0.54], *p* < 0.001). Heterogeneity among studies was high (I^2^ = 82%). Through sensitivity analysis, it was found that one study (Guo, 2019) has a great impact on heterogeneity. After excluding this study, the results show that there is no heterogeneity in the other 5 studies (I^2^ = 0%) (Figure 4).

#### Effects of Traditional Chinese Fitness Exercises on Sleep Disorder Among College Students

**Figure 5:**
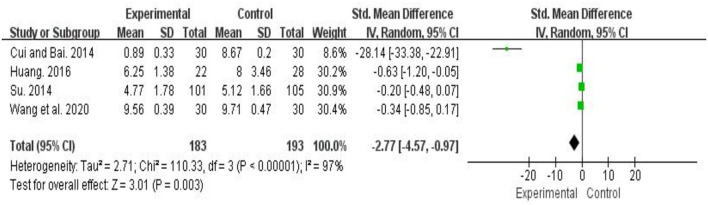
Meta-analysis of traditional fitness exercises on sleep disorder among college students.

A total 4 studies (*n* = 574) examined the effect of traditional Chinese fitness exercises on sleep disorder (Cui and Bai, 2014; Su, 2014; Huang, 2016; Wang et al., 2020). The pooled results showed that the significantly effect of traditional Chinese fitness exercises on reducing sleep disorder among college students compared to control group (SMD = −2.77, 95% *CI* [−4.57, −0.97], *p* < 0.05). Heterogeneity among studies was high (I^2^ = 97%). Through sensitivity analysis, it was found that one study (Cui and Bai, 2014) has a great impact on heterogeneity. After excluding this study, the results show that there is no heterogeneity in the other 4 studies (I^2^ = 0%) (Figure 5).

### Publication Bias

**Figure 6:**
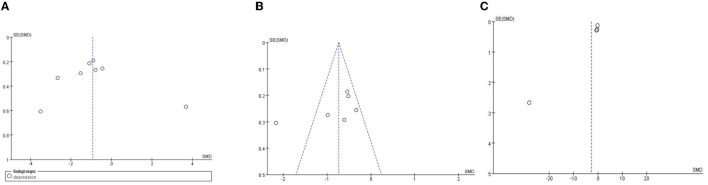
Publication bias analysis of depression **(A)**, anxiety **(B)**, sleep disorder **(C)**.

Publication bias was observed by Review Manager 5.4, and funnel plot symmetry indicates that there is no publication bias. As it is shown in Figure 6, the left and right sides of the funnel plot are basically symmetrical, most of the studies focus on the middle and upper parts of the funnel plot, but there are single studies outside of 95% *CI*, this suggests that there may be some publication bias.

## Discussion

Study, life, job, and family stressors are the common place worldwide. However, these stressors are becoming an increasingly prevalent source of psychological distress for contemporary college students. Previous studies on college students have shown that continuous mental tension and stress can lead to the onset of various negative emotions such as anxiety, depression, irritability, fear, and sleep disorders (Yang et al., 2021). Furthermore, some studies have found that sleep disorders can increase the risk of anxiety and depression (Bonnet and Arand, 2000). If the negative psychological emotions of college students are not properly alleviated, it will seriously affect the study and life of college students, and even endanger the stability of society. If the negative psychological emotions experienced by college students are not properly alleviated, they have the potential to seriously affect both the educational outcomes and life of college students. In recent years, many studies have confirmed that traditional Chinese fitness exercises can effectively alleviate college students' negative emotions and sleep disorders through the cooperation of mind and body (Wang et al., 2014a; Li et al., 2019; Wu et al., 2019). These exercises, in combination with slow breathing, aid in the relaxation of psychological pressure and cultivate spirituality; both concepts that are understood to reflect the idea of “disease prevention” in traditional Chinese medicine. Traditional Chinese fitness exercises are comprehensive mind-body exercises that have been widely applied to treat college students with negative emotions and sleep disorders. The current study systematically reviewed the previous literature and provided an objective evaluation on the effects of traditional Chinese fitness exercises on college students with negative emotions and sleep disorders.

Following the eligibility criteria, 12 RCTs comprising a total of 1,052 college students were included in this review. Although there is considerable heterogeneity within the included studies, the overall effect was positive with a statistical significance, indicating that traditional Chinese fitness exercises had an advantage over control intervention on improving negative emotions and sleep disorders of college students. Negative emotions include depression, anxiety, fear, tension, and sadness, among which depression and anxiety are the most common (Wang et al., 2020). At present, various clinical scales have been used to evaluate the degree of depression, anxiety, and sleep disorder of college students. In this review, 8 studies (Cheng et al., 2016; Liu et al., 2017; Wei et al., 2017; Yan and Wei, 2017; Chen et al., 2019; Guo, 2019; Wang et al., 2020; Zhang, 2021) showed that traditional Chinese fitness exercises had significantly effects on depression of college students compared with control group, and the difference between groups was statistically significant (*p* < 0.05), These data are consistent with the results reported by Yang et al. (2019) who showed that physical and mental exercise had significantly effects on depression.

Then, a detailed subgroup analysis based on different intervention plans, psychological health levels, and scale type found that the improvement effect of traditional Chinese fitness exercises on college students with mild to moderate psychological symptoms was superior relative to healthy controls. This result showed that the SCL-90 scale is better than the SDS scale when evaluating the improvement effect of traditional Chinese fitness exercises on depression among college students. Additionally, the results showed that five 30–60 min sessions per week for 12 weeks was the best traditional Chinese fitness exercise plan to improve the depression of college students.

In addition, six studies (Huang, 2016; Liu et al., 2017; Wei et al., 2017; Yan and Wei, 2017; Guo, 2019; Zhang, 2021) that investigated the effect of Chinese traditional fitness exercises on anxiety experienced by college students showed that the exercise group reported significantly less anxiety relative to the control group. These results were consistent with the findings of Hua and Sun (2021) who showed that the significantly effects of physical and mental exercises on anxiety, depression, and stress in college students. However, due to the limited number of studies in each group and the relatively poor study quality, the reanalyzed results may not be accurate and should be treated with caution.

Recent studies have shown that traditional Chinese fitness exercises can effectively reduce sleep disorders, thus highlighting their use as an alternative or in combination with existing therapies to treat disorders of this type (Wang et al., 2014b). Four studies (Cui and Bai, 2014; Su, 2014; Huang, 2016; Wang et al., 2020) on the effect of Chinese traditional fitness exercise on sleep disorders showed that traditional Chinese fitness exercises had significantly effect on sleep disorders of college students compared with control group, and the difference between groups was statistically significant (*p* < 0.05). It is hypothesized that these improvements may be due to the alleviation of brain fatigue and tension through the reduction of negative emotions via routine traditional Chinese fitness practice; the routine use of which promotes habits that are beneficial for the improvement of physical and psychological health (Baron et al., 2013; Yeung et al., 2018). Together these data show that in college students, despite considerable heterogeneity within the included studies, traditional Chinese fitness exercises have a significant ability to improve the onset and effect of negative emotions and sleep disorders to promote healthy wellbeing.

Meta-analysis studies are highly observational and may be affected by various factors such as bias (Wang et al., 2014b). Thus, this review has some limitations that should be carefully considered. Firstly, as the intervention measure assessed was traditional Chinese fitness exercises, no studies carried out outside of China and were published in English were included in this review; therefore, some studies could have been missed. Furthermore, as this meta-analysis was limited to Chinese college students, it is possible that our findings may not reflect the global sociodemographic distribution of all students and their relationship to stress as well the ability of traditional Chinese exercises to alleviate it. Secondly, the 12 RCTs included in the study are not detailed enough in terms of their method of randomization, allocation concealment, blind method setting, outcome indicators, and loss of follow-up reports – all of which may be due to the low quality of the original literature. Thirdly, due to the small number of studies included in this review, it is impossible to robustly analyze all relevant indicators. Additionally, due to the small sample size, there exists a relatively high risk of bias and heterogeneity that are unable to overcome relatively weak methodologies. Lastly, studies that found neutral or negative results are not often published and as such were not included in the current meta-analysis and may reduce the accuracy of our results.

## Conclusion

In conclusion, the results of this systematic review and meta-analysis demonstrated that traditional Chinese fitness exercises can improve depression, anxiety, and sleep disorders in college students relative to those who did use these practices. Future research should focus on carrying out high-quality, highly powered RCTs of college students that take into account different degrees of psychological symptoms as well as varying geographic locations. Additionally, exercise intensity should also be included as a quantifiable parameter to assess the effectiveness of different traditional Chinese exercises.

## Data Availability Statement

The datasets presented in this study can be found in online repositories. The names of the repository/repositories and accession number(s) can be found in the article/Supplementary Material.

## Author Contributions

TY: conceptualization, formal analysis, and writing—original draft. YG and YC: formal analysis and writing—review and editing. YZ and YC: screening and formal analysis. YG, YZ, and YC: writing—review and editing. All authors contributed to the article and approved the submitted version.

## Funding

This work was partially supported by Scientific Research Funding Project of Liaoning Provincial Education Department (WJC2020ST07) and Science and Technology Research Project of Fitness Qigong Management Center of General Administration of Sport of China (QG2018039).

## Conflict of Interest

The authors declare that the research was conducted in the absence of any commercial or financial relationships that could be construed as a potential conflict of interest.

## Publisher's Note

All claims expressed in this article are solely those of the authors and do not necessarily represent those of their affiliated organizations, or those of the publisher, the editors and the reviewers. Any product that may be evaluated in this article, or claim that may be made by its manufacturer, is not guaranteed or endorsed by the publisher.
