# Supplementary material for: Effects of Traditional Chinese Fitness Exercises on Negative Emotions and Sleep Disorders in College Students: A Systematic Review and Meta-Analysis
Source: Front Psychol. 2022 Jul 4;13:908041. doi: 10.3389/fpsyg.2022.908041 (PMC9291289; doi:10.3389/fpsyg.2022.908041)
Supplement: Supplementary file 1 [file Table_1.docx]

# Search strategies

1. **PubMed**

#1. ("anxiety"[MeSH Terms] OR "anxiety"[All Fields] OR "anxieties"[All Fields] OR "anxiety s"[All Fields] OR ("anxiety"[MeSH Terms] OR "anxiety"[All Fields] OR "nervousness"[All Fields] OR ("anxiety"[MeSH Terms] OR "anxiety"[All Fields] OR "anxiousness"[All Fields]) OR ("anxiety"[MeSH Terms] OR "anxiety"[All Fields] OR "anxieties"[All Fields] OR "anxiety s"[All Fields])

#2. ("depressed"[All Fields] OR "depression"[MeSH Terms] OR "depression"[All Fields] OR "depressions"[All Fields] OR "depression s"[All Fields] OR "depressive disorder"[MeSH Terms] OR ("depressive"[All Fields] AND "disorder"[All Fields]) OR "depressive disorder"[All Fields] OR "depressivity"[All Fields] OR "depressive"[All Fields] OR "depressively"[All Fields] OR "depressiveness"[All Fields] OR "depressives"[All Fields] OR ("depression"[MeSH Terms] OR "depression"[All Fields] OR ("depressive"[All Fields] AND "symptoms"[All Fields]) OR "depressive symptoms"[All Fields] OR ("depressed"[All Fields] OR "depression"[MeSH Terms] OR "depression"[All Fields] OR "depressions"[All Fields] OR "depression s"[All Fields] OR "depressive disorder"[MeSH Terms] OR ("depressive"[All Fields] AND "disorder"[All Fields]) OR "depressive disorder"[All Fields] OR "depressivity"[All Fields] OR "depressive"[All Fields] OR "depressively"[All Fields] OR "depressiveness"[All Fields] OR "depressives"[All Fields]) OR ("depression"[MeSH Terms] OR "depression"[All Fields] OR ("emotional"[All Fields] AND "depression"[All Fields]) OR "emotional depression"[All Fields])

#3. ("emotional regulation"[MeSH Terms] OR ("emotional"[All Fields] AND "regulation"[All Fields]) OR "emotional regulation"[All Fields] OR ("emotional regulation"[MeSH Terms] OR ("emotional"[All Fields] AND "regulation"[All Fields]) OR "emotional regulation"[All Fields] OR ("emotional"[All Fields] AND "regulations"[All Fields]) OR "emotional regulations"[All Fields] OR ("emotional regulation"[MeSH Terms] OR ("emotional"[All Fields] AND "regulation"[All Fields]) OR "emotional regulation"[All Fields] OR ("emotion"[All Fields] AND "self"[All Fields] AND "regulation"[All Fields]) OR "emotion self regulation"[All Fields])

#4. #1OR #2 OR #3

#5. ("qigong"[MeSH Terms] OR "qigong"[All Fields] OR ("qigong"[MeSH Terms] OR "qigong"[All Fields] OR ("qi"[All Fields] AND "gong"[All Fields]) OR "qi gong"[All Fields] OR ("qigong"[MeSH Terms] OR "qigong"[All Fields] OR ("ch i"[All Fields] AND "kung"[All Fields]) OR "ch i kung"[All Fields])) OR (("tai ji"[MeSH Terms] OR ("tai"[All Fields] AND "ji"[All Fields]) OR "tai ji"[All Fields] OR "taiji"[All Fields]) AND ("tai ji"[MeSH Terms] OR ("tai"[All Fields] AND "ji"[All Fields]) OR "tai ji"[All Fields] OR ("tai ji"[MeSH Terms] OR ("tai"[All Fields] AND "ji"[All Fields]) OR "tai ji"[All Fields] OR ("tai"[All Fields] AND "chi"[All Fields]) OR "tai chi"[All Fields]) OR ("tai ji"[MeSH Terms] OR ("tai"[All Fields] AND "ji"[All Fields]) OR "tai ji"[All Fields] OR ("chi"[All Fields] AND "tai"[All Fields]) OR "chi tai"[All Fields]) OR ("tai ji"[MeSH Terms] OR ("tai"[All Fields] AND "ji"[All Fields]) OR "tai ji"[All Fields] OR ("tai"[All Fields] AND "ji"[All Fields] AND "quan"[All Fields]) OR "tai ji quan"[All Fields]) OR ("tai ji"[MeSH Terms] OR ("tai"[All Fields] AND "ji"[All Fields]) OR "tai ji"[All Fields] OR ("ji"[All Fields] AND "quan"[All Fields] AND "tai"[All Fields]) OR "ji quan tai"[All Fields]) OR ("tai ji"[MeSH Terms] OR ("tai"[All Fields] AND "ji"[All Fields]) OR "tai ji"[All Fields] OR ("quan"[All Fields] AND "tai"[All Fields] AND "ji"[All Fields]) OR "quan tai ji"[All Fields]) OR ("tai ji"[MeSH Terms] OR ("tai"[All Fields] AND "ji"[All Fields]) OR "tai ji"[All Fields] OR "taiji"[All Fields]) OR ("tai ji"[MeSH Terms] OR ("tai"[All Fields] AND "ji"[All Fields]) OR "tai ji"[All Fields] OR "taijiquan"[All Fields]) OR ("tai ji"[MeSH Terms] OR ("tai"[All Fields] AND "ji"[All Fields]) OR "tai ji"[All Fields] OR ("t ai"[All Fields] AND "chi"[All Fields]) OR "t ai chi"[All Fields]) OR ("tai ji"[MeSH Terms] OR ("tai"[All Fields] AND "ji"[All Fields]) OR "tai ji"[All Fields] OR ("tai"[All Fields] AND "chi"[All Fields] AND "chuan"[All Fields]) OR "tai chi chuan"[All Fields])

#6. ("random allocation"[MeSH Terms] OR ("random"[All Fields] AND "allocation"[All Fields]) OR "random allocation"[All Fields] OR "random"[All Fields] OR "randomization"[All Fields] OR "randomized"[All Fields] OR "randomisation"[All Fields] OR "randomisations"[All Fields] OR "randomise"[All Fields] OR "randomised"[All Fields] OR "randomising"[All Fields] OR "randomizations"[All Fields] OR "randomize"[All Fields] OR "randomizes"[All Fields] OR "randomizing"[All Fields] OR "randomness"[All Fields] OR "randoms"[All Fields] OR (("random allocation"[MeSH Terms] OR ("random"[All Fields] AND "allocation"[All Fields]) OR "random allocation"[All Fields] OR "random"[All Fields] OR "randomization"[All Fields] OR "randomized"[All Fields] OR "randomisation"[All Fields] OR "randomisations"[All Fields] OR "randomise"[All Fields] OR "randomised"[All Fields] OR "randomising"[All Fields] OR "randomizations"[All Fields] OR "randomize"[All Fields] OR "randomizes"[All Fields] OR "randomizing"[All Fields] OR "randomness"[All Fields] OR "randoms"[All Fields]) AND "controlled"[All Fields] AND "tria"[All Fields])

#7. #4 AND #5 AND #6

1. **EMbase**

(('anxiety'/exp OR ('nervousness':ab,ti OR 'anxiousnes' OR 'anxieties')) OR ('depression'/exp OR ('depressive symptoms':ab,ti OR 'depressive' OR 'emotional depression')) OR ('emotion regulation'/exp OR ('emotional regulations':ab,ti OR 'emotion elf-regulation' OR 'emotional regulation'))) AND ((qigong OR ('qi gong':ab,ti OR 'chi kung')) OR ('tai chi'/exp OR (('tai-ji':ab,ti OR 'taiji' OR 'chi, tai':ab,ti OR 'tai ji quan':ab,ti OR 'ji quan, tai' OR 'quan, tai ji') AND 'taijiquan':ab,ti OR 'tai chi chuan'))) AND 'randomized controlled trial'/exp

1. **Cochrane library**

| Search | Query | Items found |
| --- | --- | --- |
| #1 | MeSH descriptor: [Anxiety] explode all trees | 8902 |
| #2 | (Nervousness):ti,ab,kw OR (Anxiousnes):ti,ab,kw OR (Anxieties):ti,ab,kw | 84461 |
| #3 | #1 OR #2 | 84593 |
| #4 | MeSH descriptor: [Depression] explode all trees | 13714 |
| #5 | (Depressive Symptoms):ti,ab,kw OR (Depressive):ti,ab,kw OR (Emotional Depression):ti,ab,kw | 95856 |
| #6 | #4 OR #5 | 95856 |
| #7 | MeSH descriptor: [Emotional Regulation] explode all trees | 88 |
| #8 | (Emotional Regulations):ti,ab,kw OR (Emotion Self-Regulation):ti,ab,kw OR (Emotional Regulation):ti,ab,kw | 3743 |
| #9 | #7 OR #8 | 3743 |
| #10 | #3 OR #6 OR #9 | 149000 |
| #11 | MeSH descriptor: [Qigong] explode all trees | 90 |
| #12 | (Qi Gong):ti,ab,kw OR (Ch'i Kung):ti,ab,kw | 65 |
| #13 | #11 OR #12 | 151 |
| #14 | MeSH descriptor: [Tai Ji] explode all trees | 396 |
| #15 | (Tai-ji):ti,ab,kw OR (taijiquan):ti,ab,kw OR (Tai Ji Quan):ti,ab,kw OR (Tai Chi Chuan):ti,ab,kw OR (Quan, Tai Ji):ti,ab,kw | 599 |
| #16 | #14 OR #15 | 599 |
| #17 | #13 OR #16 | 732 |
| #18 | #10 AND #17 | 138 |

1. **Scopus**

( TITLE-ABS-KEY ( depressed ) OR TITLE-ABS-KEY ( depression ) OR TITLE-ABS-KEY ( depressions ) OR TITLE-ABS-KEY ( depressive ) OR TITLE-ABS-KEY ( anxiety ) OR TITLE-ABS-KEY ( anxiousness ) OR TITLE-ABS-KEY ( anxieties ) OR TITLE-ABS-KEY ( emotional ) OR TITLE-ABS-KEY ( depressions ) OR TITLE-ABS-KEY ( emotion ) AND TITLE-ABS-KEY ( qigong ) OR TITLE-ABS-KEY ( qi AND gong ) OR TITLE-ABS-KEY ( tai AND chi AND kung ) OR TITLE-ABS-KEY ( tai AND ji ) OR TITLE-ABS-KEY ( taiji ) OR TITLE-ABS-KEY ( tai AND chi ) OR TITLE-ABS-KEY ( tai AND ji AND quan ) OR TITLE-ABS-KEY ( tai AND chi AND chuan ) OR TITLE-ABS-KEY (Baduanjin ) OR TITLE-ABS-KEY ( Yijinjing ) OR TITLE-ABS-KEY ( Wuqinxi ) OR TITLE-ABS-KEY ( Liuzijue) OR TITLE-ABS-KEY (Zhanzhuanggong) AND TITLE-ABS-KEY ( random AND allocation ) OR TITLE-ABS-KEY ( random ) OR TITLE-ABS-KEY ( randomization ) OR TITLE-ABS-KEY ( randomized ) OR TITLE-ABS-KEY ( randomisation ) OR TITLE-ABS-KEY ( randomise ) OR TITLE-ABS-KEY ( trial ) OR TITLE-ABS-KEY ( random AND trial ) OR TITLE-ABS-KEY ( rct ) AND TITLE-ABS-KEY ( college AND students ) OR TITLE-ABS-KEY ( college ) OR TITLE-ABS-KEY ( university ) )

1. **EBSCO**

AB ( AB depressed OR AB depression OR AB depressions OR AB depressive disorder OR AB anxiety OR AB anxiousness OR AB anxiety symptoms OR AB emotional regulation OR AB emotional OR AB depressions OR AB emotion ) AND AB ( AB qigong OR AB qi gong OR AB tai chi kung OR AB tai ji OR AB taiji OR AB tai chi OR AB tai chi chuan OR AB Baduanjin OR AB Yijinjing OR AB Wuqinxi OR AB Liuzijue OR AB Zhanzhuanggong ) AND AB ( AB random allocation OR AB random OR AB randomization OR AB randomized OR AB randomisation OR AB randomise OR AB trial OR AB random trial ) AND AB ( College students OR AB college OR AB university )
